# Genomic loss of HLA alleles may affect the clinical outcome in low-risk myelodysplastic syndrome patients

**DOI:** 10.18632/oncotarget.26405

**Published:** 2018-12-11

**Authors:** Paola Montes, Martin Kerick, Mónica Bernal, Francisca Hernández, Pilar Jiménez, Pilar Garrido, Ana Márquez, Manuel Jurado, Javier Martin, Federico Garrido, Francisco Ruiz-Cabello

**Affiliations:** ^1^ Servicio de Análisis Clínicos e Inmunología, UGC de Laboratorio Clínico, Hospital Universitario Virgen de las Nieves, Granada, Spain; ^2^ Instituto de Parasitología y Biomedicina López Neyra, CSIC, Granada, Spain; ^3^ UGC de Hematología, Hospital Universitario Virgen de las Nieves, Granada, Spain; ^4^ Instituto de Investigación Biosanitaria ibs.Granada, Granada, Spain; ^5^ Departamento Bioquímica, Biología Molecular e Inmunología III, Universidad de Granada, Granada, Spain

**Keywords:** human leukocyte antigen (HLA), loss of heterozygosity (LOH), single nucleotide polymorphism array (SNP array), myelodysplastic syndrome (MDS), hematopoietic stem cell transplantation (HSCT)

## Abstract

The Revised International Prognostic Score and some somatic mutations in myelodysplastic syndrome (MDS) are independently associated with transformation to acute myeloid leukemia (AML). Immunity has also been implicated in the pathogenesis of MDS, although the underlying mechanism remains unclear. We performed a SNP array on chromosome 6 in CD34^+^ purified blasts from 19 patients diagnosed with advanced MDS and 8 patients with other myeloid malignancies to evaluate the presence of loss of heterozygosity (LOH) in HLA and its impact on disease progression. Three patients had acquired copy-neutral LOH (CN-LOH) on 6p arms, which may disrupt antigen presentation and act as a mechanism for immune system evasion. Interestingly, these patients had previously been classified at low risk of AML progression, and the poor outcome cannot be explained by the acquisition of adverse mutations. LOH HLA was not detected in the remaining 24 patients, who all had adverse risk factors. In summary, the clinical outcome of patients with advanced MDS might be influenced by HLA allelic loss, wich allows subclonal expansions to evade cytotoxic-T and NK cell attack. CN-LOH HLA may therefore be a factor favoring MDS progression to AML independently of the somatic tumor mutation load.

## INTRODUCTION

Myelodysplastic Syndromes (MDS) are a range of heterogeneous clonal hematologic diseases characterized by ineffective hematopoiesis and a tendency to develop acute myeloid leukemia (AML) [1]. Given the heterogeneity of the disease, several prognostic scoring systems are currently used to stratify patients according to the risk of AML development, including the International Prognostic Scoring System (IPSS) and the revised IPSS (IPSS-R), which incorporates a cytogenetic risk classification [2, 3]. Recurrent genetic alterations are observed in most patients with MDS, although only half of them have an altered karyotype. The most frequently mutated genes in MDS patients are involved in RNA splicing (*SF3B1, SRSF2, U2AF1, ZRSR2*) and in epigenetic regulation of DNA, including methylation (*TET2, DNMT3A, IDH1/2*) and chromatin regulation (*ASXL1, EZH2)* processes. Mutations of genes that participate in cellular signaling pathways (*FLT3, NRAS*) are less frequent in these patients and are acquired at later stages of disease progression [4–6]. In addition, markers of high molecular risk (*TP53, EZH2, ETV6, RUNX1, ASXL1, SRSF2*) have been defined that predict worse overall survival and a greater risk of leukemic transformation and post-transplantation relapse, independently of prognostic scores, whereas mutations in *SF3B1* have been associated with improved survival outcomes [7–9].

Dysregulation of the immune system also appears to be implicated in the pathogenesis of MDS, although most studies have focused on the role of the tumor microenvironment [10, 11]. Immune evasion is a hallmark of cancer [12–15], and one of the main escape mechanisms is thought to be a reduction in antigen presentation due to HLA class I (HLA-I) abnormality. Although total lack of HLA-I antigen expression is frequent in tumor tissue, it is rarely observed in leukemia at presentation [16–18]. Limited research has been conducted on HLA-I antigen expression in hematologic malignancies such as B-cell and Hodgkin lymphoma [19, 20], chronic lymphoblastic leukemia (CLL) [21], acute lymphoblastic leukemia (ALL), and acute myeloid leukemia (AML) [22–24]. Copy neutral loss of heterozygosity (CN-LOH) in the HLA region has been described in approximately 13% of aplastic anemia patients as a possible mechanism to escape the autoimmune response of cytotoxic T-CD8 lymphocytes (CTLs) [25, 26]. This mechanism has also been reported in a significant proportion of AML patients who relapsed after donor-lymphocyte infusions following transplantation with hematopoietic stem cells from haploidentical donors [24, 27, 28]. It is likely that selective rather than total loss contributes more effectively to simultaneous escape from T and NK cells [13, 29]. Haplotype loss is a frequent signature in various human tumors and is particularly relevant in non-small cell lung cancer (NSCLC) [30, 31]. These data have been reported in other studies, which suggest that the high prevalence of LOH HLA is attributable to positive selection during tumor evolution, facilitating immune escape [32]. In the present study, single nucleotide polymorphism (SNP) array techniques were used to explore the contribution of the LOH HLA mechanism to MDS progression. Extensive 6p LOH, including the complete HLA region, may be an immune escape mechanism, explaining its impact on clonal evolution and disease progression.

## RESULTS

### Patient characteristics

The incidence of MDS in Spain is estimated at 4-5 cases per 100,000 persons/year [1]. Over the past 4 years, 120 new cases have been recorded in our geographical area. The present study includes 19 of these cases, including 8 cases of advanced MDS with excess blasts (MDS EB) and 11 cases of AML secondary to MDS (sAML).

Of the total of 27 patients included in the study, in 14 of the 21 patients with MDS, sAML, or CMML, the baseline IPSS-R score was Very High Risk (VHR, n= 6), High Risk (HR, n=1), Intermediate (n=3), or Low/Very Low Risk (LR or VLR, n=4); no data were available for 7 patients. The cytogenetic risk score was very poor (n=6), intermediate (n=4), or good/very good (n=5); no data were available for 6 patients (Table 1).

**Table 1:** Characteristics of patients included in the study

PATIENTS	% BLASTS in BM	WHO-2016	TIME FROM DIAGNOSIS TO ANALYSIS	IPSS-R	IPSS-R (Score)	CYTOGENETIC SCORE	MUTATIONAL PROFILE	TREATMENT
**1**	7	MDS EB-1	<1 month	VHR	6	Very Poor	***TP53****, U2AF1, TET2*	Azacitidine
**2**	12	MSD EB-2	<1 month	VHR	6	Very Poor	***TP53***	Azacitidine
**3**	11	MDS EB-2	1.5 years	VHR	7	Intermediate	ND	Azacitidine
**4**	12	MSD EB-2	6.5 years	INT	4,5	Intermediate	***RUNX1***	Azacitidine
**5**	13	MSD-EB-2	<1 month	VHR	8,5	Very Poor	Not detected	Supportive care
**6**	10	MSD EB-2	ND	ND	ND	ND	***ASXL1****,* ***RUNX1****, SRSF2, IDH1, TET2*	ND
**7**	10	MSD EB-2	<1 month	VHR	8,5	Very Poor	***TP53***	RIC-Allo-HSCT
**8**	10	MSD EB-2	<1 month	INT	3	Intermediate	*SF3B1, NPM1*	RIC-Allo-HSCT
**9**	20	sAML	3 years	LR	2	Good	Not detected	Lenalidomide
**10**	22	sAML	2 years	LR	2	Good	*SF3B1*	RIC-Allo-HSCT
**11**	34	sAML	<1 month	ND	ND	Very Poor	***TP53***	Azacitidine
**12**	40	sAML	1 year	INT	3	Good	*SF3B1, DNMT3A, TET2*	Azacitidine
**13**	45	sAML	2 years	VHR	6,5	Intermediate	***ASXL1, EZH2, ETV6****, KRAS, PTPN11, ABL1*	Azacitidine
**14**	30	sAML	1.5 years	HR	5	ND	***TP53****, U2AF1, NRAS*	Azacitidine
**15**	25	sAML	ND	ND	ND	Good	***RUNX1****, U2AF1, BCOR, NRAS, FLT3-TKD*	Azacitidine
**16**	40	sAML	ND	ND	ND	ND	***RUNX1, EZH2, ASXL1****, SF3B1, SETBP1*	Azacitidine
**17**	57	sAML	ND	ND	ND	ND	***RUNX1****, SF3B1, IDH2, DNMT3A*	Azacitidine
**18**	20	sAML	ND	ND	ND	ND	***TP53***	Azacitidine
**19**	86	sAML	ND	ND	ND	ND	***RUNX1, EZH2****, IDH2, CUX1, STAG2*	Azacitidine
**20**^a^	1	CMML-0	1.5 years	VL	1	Good	***ASXL1****, SRSF2, TET2*	Supportive care
**21**^a^	2	CMML-1	2 years	LR	2	Very Poor	***TP53****, KRAS*	Azacitidine
**22**	99	*De novo* AML	3 years	NA	NA	Good	*NPM1, FLT3-TKD, WT1*	RIC-Allo-HSCT
**23**	26	*De novo* AML	<1 month	NA	NA	ND	Not detected	Chemotherapy
**24**	70	*De novo* AML	<1 month	NA	NA	ND	*NPM1, PTPN11*	Chemotherapy
**25**	56	*De novo* AML	<1 month	NA	NA	ND	*ZRSR2*	Chemotherapy
**26**	70	*De novo* AML	<1 month	NA	NA	Good	*DNMT3A*	Chemotherapy
**27**	20	*De novo* AML	<1 month	NA	NA	ND	***TP53***	Chemotherapy

Note: BM: Bone Marrow; MDS EB-1, -2: Myelodysplastic Syndrome with Excess of Blasts-1, -2; sAML: Acute Myeloid Leukemia secondary to MDS; CMML-0: Chronic Myelomonocytic Leukemia with <5% blasts in BM; CMML-1: Chronic Myelomonocytic Leukemia with 5-9% blasts in BM.

IPSS-R: Revised International Prognostic Scoring System; VHR: Very High Risk; HR: High Risk, INT: Intermediate, LR: Low Risk; VL: Very Low Risk.

NA: Not Applicable; ND: No Data.

RIC-Allo-HSCT: Reduced Intensity Conditioning Allogeneic Hematopoietic Stem Cell Transplantation.

In Mutational Profile, genes indicated in bold are High Molecular Risk (HMR) genes [7].

^a^Patient 20 and 21, DNA used in analysis was obtained from CD14^+^ monocytes (>40% monocytes in BM).

Four patients (2 MDS-EB, 1 sAML, and 1 *de novo* AML) underwent reduced intensity conditioning allogeneic hematopoietic stem cell transplantation (RIC-Allo-HSCT). The remaining patients received 5-azacitidine (n=14), lenalidomide (n=1), induction chemotherapy (n=5), or supportive care (n=2); no data were available for one patient (Table 1).

### Mutational analysis

Next, the mutational profile of the patients was analyzed, sequencing target regions of 54 genes associated with myeloid neoplasms using NGS techniques. One patient (Patient 3) could not be studied by this procedure. Out of the 26 patients studied, 23 (85.2%) had mutations in driver genes (Table 1 and Supplementary Table 1). Most mutant driver genes were splicing genes (*SF3B1, U2AF1, SRSF2*), methylation genes (*TET2, IDH1/2, DNMT3A*), and/or chromatin regulation genes (*ASXL1, EZH2*). At least one of the aforementioned genes was mutated in 15 of the 26 patients (57.7%). There was also a notable frequency of mutations affecting *RUNX1* (n=6) and *TP53* (n=8). The majority of patients with mutations in *TP53* (5 out of 8 patients) had no alterations in other genes.

Among the 20 patients with MDS-EB, sAML, or CMML, 8 (40%) (4 MDS-EB, 3 sAML and 1 CMML) had ≤2 mutations in driver genes, 10 (50%) (2 MDS-EB, 7 sAML and 1 CMML) had ≥3 mutations, and only 2 (10%) (1 MDS-EB and 1 sAML) had no mutations in the sequenced genes. Among the 6 patients with *de novo* AML group (n=6), 4 had ≤2 mutations in driver genes. Only patient 22 had 3 mutations, while patient 23 had no mutations in the studied genes. Hence, the patients with sAML had a larger number of mutations affecting driver genes in comparison to patients with MDS-EB or *de novo* AML.

Furthermore, 23 of the 26 patients had a mutation with allelic frequency (Variant Allele Frequency, VAF) ≥40% in at least one driver gene, and only 3 patients (1 sAML and 2 de novo AML) had VAF<40% in mutated genes (Supplementary Table 1). In addition, 13 (72%) of the 18 patients with MDS-EB or sAML had at least one mutation in a High-Molecular-Risk (HMR) gene (*TP53, RUNX1, ASXL1, ETV6, EZH2*). Interestingly, *SF3B1* mutation, considered a good prognosis factor, was observed in three of the five patients with no HMR gene mutation (Table 1 and Supplementary Table 1).

### LOH analysis in HLA region of chromosome 6

We performed SNP array studies of chromosome 6 to analyze LOH in the HLA region (6p21) (LOH HLA) using DNA from purified CD34^+^ blasts and DNA from autologous CD3^+^ control cells. In 24 (89%) out of the 27 patients studied, no alterations were detected on chromosome 6 in the CD34^+^ cell fraction in comparison to control cells (data not shown). These results were confirmed by HLA typing techniques using Luminex technology. The purified CD34^+^ cell fraction of the 24 patients was found to retain the two HLA haplotypes observed in the autologous control cells (Table 2). Among the 24 patients with no alterations on chromosome 6, 17 had MDS or sAML with high-risk IPSS-R scores and/or mutations affecting HMR genes, while 2 of the remaining 7 patients had CMML and 5 had *de novo* AMLs.

**Table 2:** Genomic typing HLA alleles

Patient	Sample	HLA-A		HLA-B		HLA-C		HLA-DRB1		HLA-DQB1	
**1**	#1	A*03:01	A*26:01	B*07:02	B*35:01	C*03:03	C*07:02	DRB1*15:01	DRB1*15:01	DQB1*05:02	DQB1*06:02
	#2	A*03:01	A*26:01	B*07:02	B*35:01	C*03:03	C*07:02	DRB1*15:01	DRB1*15:01	DQB1*05:02	DQB1*06:02
**2**	#1	A*11:01	A*31:01	B*13:02	B*51:01	C*06:02	C*15:02	DRB1*07:01	DRB1*08:01	DQB1*02:02	DQB1*04:02
	#2	A*11:01	A*31:01	B*13:02	B*51:01	C*06:02	C*15:02	DRB1*07:01	DRB1*08:01	DQB1*02:02	DQB1*04:02
**3**	#1	A*24:02	A*32:01	B*40:06	B*52:01	C*12:02	C*15:02	DRB1*04:07	DRB1*13:02	DQB1*03:01	DQB1*06:04
	#2	A*24:02	A*32:01	B*40:06	B*52:01	C*12:02	C*15:02	DRB1*04:07	DRB1*13:02	DQB1*03:01	DQB1*06:04
**4**	#1	A*02:02	A*13:01	B*07:02	B*15:16	C*07:02	C*14:02	DRB1*01:03	DRB1*07:01	DQB1*02:02	DQB1*05:01
	#2	A*02:02	A*13:01	B*07:02	B*15:16	C*07:02	C*14:02	DRB1*01:03	DRB1*07:01	DQB1*02:02	DQB1*05:01
**5**	#1	A*02:01	A*02:02	B*44:02	B*58:01	C*05:01	C*07:01	DRB1*04:05	DRB1*11:01	DQB1*03:01	DQB1*03:01
	#2	A*02:01	A*02:02	B*44:02	B*58:01	C*05:01	C*07:01	DRB1*04:05	DRB1*11:01	DQB1*03:01	DQB1*03:01
**6**	#1	A*01:01	A*24:02	B*13:02	B*44:02	C*05:01	C*06:02	DRB1*03:01	DRB1*04:08	DQB1*02:01	DQB1*03:01
	#2	A*01:01	A*24:02	B*13:02	B*44:02	C*05:01	C*06:02	DRB1*03:01	DRB1*04:08	DQB1*02:01	DQB1*03:01
**7**	#1	A*01:01	A*03:01	B*35:08	B*44:03	C*04:01	C*16:01	DRB1*07:01	DRB1*11:01	DQB1*05:01	DQB1*06:04
	#2	A*01:01	A*03:01	B*35:08	B*44:03	C*04:01	C*16:01	DRB1*07:01	DRB1*11:01	DQB1*05:01	DQB1*06:04
**8**	#1	A*02:01	A*02:01	B*35:03	B*44:03	C*04:01	C*04:01	DRB1*07:01	DRB1*07:01	DQB1*02:02	DQB1*02:02
	#2	A*02:01	A*02:01	B*35:03	B*44:03	C*04:01	C*04:01	DRB1*07:01	DRB1*07:01	DQB1*02:02	DQB1*02:02
**9**	#1	A*30:02	−	B*18:01	−	C*05:01	−	DRB1*03:01	−	DQB1*02:01	−
	#2	A*30:02	A*66:01	B*18:01	B*51:01	C*05:01	C*07:01	DRB1*03:01	DRB1*13:03	DQB1*02:01	DQB1*03:01
**10**	#1	A*02:01	−	−	B*18:01	C*07:01	−	−	DRB1*11:04	DQB1*03:01	−
	#2	A*02:01	A*02:01	B*39:01	B*18:01	C*07:01	C*12:03	DRB1*11:01	DRB1*11:04	DQB1*03:01	DQB1*03:01
**11**	#1	A*03:01	A*32:01	B*15:01	B*52:01	C*03:03	C*12:02	DRB1*04:04	DRB1*15:02	DQB1*03:02	DQB1*06:01
	#2	A*03:01	A*32:01	B*15:01	B*52:01	C*03:03	C*12:02	DRB1*04:04	DRB1*15:02	DQB1*03:02	DQB1*06:01
**12**	#1	A*30:02	A*33:01	B*14:02	B*40:01	C*03:04	C*08:02	DRB1*04:04	DRB1*13:03	DQB1*03:01	DQB1*03:02
	#2	A*30:02	A*33:01	B*14:02	B*40:01	C*03:04	C*08:02	DRB1*04:04	DRB1*13:03	DQB1*03:01	DQB1*03:02
**13**	#1	A*01:01	A*02:01	B*18:01	B*39:01	C*05:01	C*12:03	DRB1*14:01	DRB1*15:01	DQB1*05:03	DQB1*06:02
	#2	A*01:01	A*02:01	B*18:01	B*39:01	C*05:01	C*12:03	DRB1*14:01	DRB1*15:01	DQB1*05:03	DQB1*06:02
**14**	#1	A*01:01	A*31:01	B*08:01	B*53:01	C*04:01	C*07:01	DRB1*01:01	DRB1*03:01	DQB1*02:01	DQB1*05:01
	#2	A*01:01	A*32:01	B*08:01	B*53:01	C*04:01	C*07:01	DRB1*01:01	DRB1*03:01	DQB1*02:01	DQB1*05:01
**15**	#1	A*25:01	A*30:02	B*18:01	B*44:02	C*05:01	C*05:01	DRB1*10:01	DRB1*13:01	DQB1*05:01	DQB1*06:03
	#2	A*25:01	A*30:02	B*18:01	B*44:02	C*05:01	C*05:01	DRB1*10:01	DRB1*13:01	DQB1*05:01	DQB1*06:03
**16**	#1	A*24:02	A*30:02	B*18:01	B*50:01	C*05:01	C*06:02	DRB1*03:01	DRB1*04:04	DQB1*02:01	DQB1*04:02
	#2	A*24:02	A*30:02	B*18:01	B*50:01	C*05:01	C*06:02	DRB1*03:01	DRB1*04:04	DQB1*02:01	DQB1*04:02
**17**	#1	A*02:01	A*02:05	B*18:01	B*50:01	C*05:01	C*06:02	DRB1*03:01	DRB1*11:01	DQB1*02:01	DQB1*03:01
	#2	A*02:01	A*02:05	B*18:01	B*50:01	C*05:01	C*06:02	DRB1*03:01	DRB1*11:01	DQB1*02:01	DQB1*03:01
**18**	#1	A*02:05	A*31:01	B*14:01	B*58:01	C*07:01	C*08:02	DRB1*03:01	DRB1*04:05	DQB1*02:01	DQB1*03:02
	#2	A*02:05	A*31:01	B*14:01	B*58:01	C*07:01	C*08:02	DRB1*03:01	DRB1*04:05	DQB1*02:01	DQB1*03:02
**19**	#1	A*29:02	A*33:03	B*44:02	B*44:03	C*05:01	C*16:01	DRB1*01:01	DRB1*12:01	DQB1*03:01	DQB1*05:01
	#2	A*29:02	A*33:03	B*44:02	B*44:03	C*05:01	C*16:01	DRB1*01:01	DRB1*12:01	DQB1*03:01	DQB1*05:01
20*	#1	A*24:02	A*29:02	B*07:02	B*44:03	C*07:02	C*16:01	DRB1*07:01	DRB1*15:01	DQB1*02:02	DQB1*06:02
	#2	A*24:02	A*29:02	B*07:02	B*44:03	C*07:02	C*16:01	DRB1*07:01	DRB1*15:01	DQB1*02:02	DQB1*06:02
21*	#1	A*01:01	A*11:01	B*35:02	B*53:01	C*04:01	C*04:01	DRB1*13:01	DRB1*13:02	DQB1*06:04	DQB1*06:03
	#2	A*01:01	A*11:01	B*35:02	B*53:01	C*04:01	C*04:01	DRB1*13:01	DRB1*13:02	DQB1*06:04	DQB1*06:03
**22**	#1	−	A*26:01	−	B*38:01	−	C*12:03	DRB1*01:03	−	DQB1*03:02	−
	#2	A*02:01	A*26:01	B*07:02	B*38:01	C*07:02	C*12:03	DRB1*01:03	DRB1*04:02	DQB1*03:02	DQB1*05:01
**23**	#1	A*02:01	A*66:01	B*51:05	B*41:02	C*04:01	C*17:01	DRB1*04:03	DRB1*04:04	DQB1*03:01	DQB1*06:03
	#2	A*02:01	A*66:01	B*51:05	B*41:02	C*04:01	C*17:01	DRB1*04:03	DRB1*04:04	DQB1*03:01	DQB1*06:03
**24**	#1	A*11:01	A*11:01	B*27:05	B*35:01	C*01:02	C*04:01	DRB1*01:01	DRB1*14:01	DQB1*05:01	DQB1*05:03
	#2	A*11:01	A*11:01	B*27:05	B*35:01	C*01:02	C*04:01	DRB1*01:01	DRB1*14:01	DQB1*05:01	DQB1*05:03
**25**	#1	A*03:01	A*31:01	B*35:03	B*38:01	C*04:01	C*12:03	DRB1*13:01	DRB1*16:01	DQB1*05:02	DQB1*06:03
	#2	A*03:01	A*31:01	B*35:03	B*38:01	C*04:01	C*12:03	DRB1*13:01	DRB1*16:01	DQB1*05:02	DQB1*06:03
**26**	#1	A*24:02	A*32:01	B*35:03	B*35:08	C*04:01	C*04:01	DRB1*11:01	DRB1*13:02	DQB1*03:01	DQB1*06:09
	#2	A*24:02	A*32:01	B*35:03	B*35:08	C*04:01	C*04:01	DRB1*11:01	DRB1*13:02	DQB1*03:01	DQB1*06:09
**27**	#1	A*26:01	A*66:01	B*41:02	B*44:03	C*16:01	C*17:01	DRB1*03:01	DRB1*07:01	DQB1*02:01	DQB1*02:02
	#2	A*26:01	A*66:01	B*41:02	B*44:03	C*16:01	C*17:01	DRB1*03:01	DRB1*07:01	DQB1*02:01	DQB1*02:02

Note: HLA Luminex typing of CD34+ leukemic blasts (#1) and autologous CD3+ T-cells (#2) in samples from 27 patients in the study.

*Patients 20 and 21, DNA used in analysis was obtained from CD14+ monocytes (Sample #1).

− : Non-detected/assigned allele. Red boxes indicate the three patients in which HLA haplotype loss was detected.

LOH HLA was detected in 3 (11%) of the 27 patients (2 AML secondary to MDS isolated del(5q) and 1 *de novo* AML), as detailed below.

**Figure 1:**
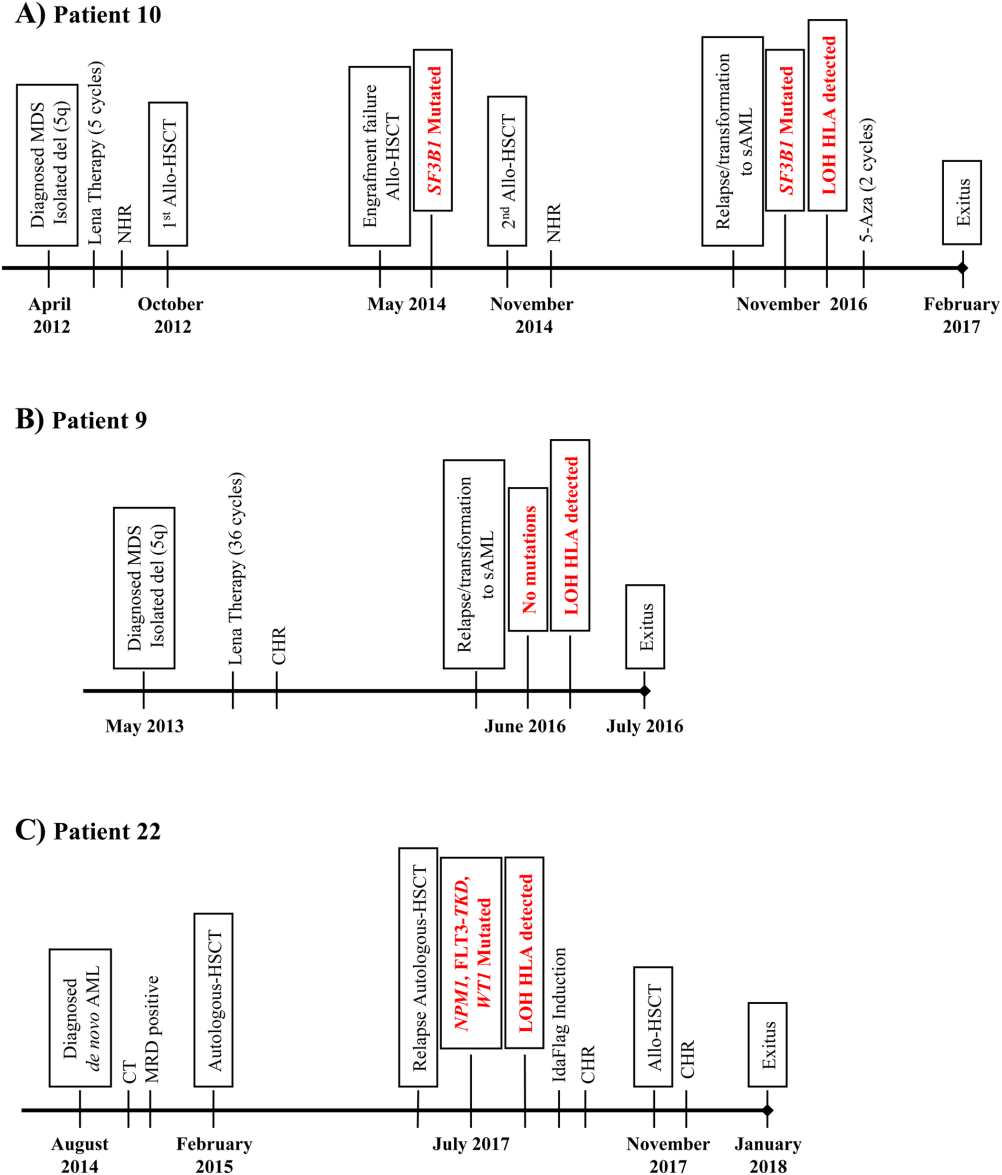
Graphic representation of the clinical progression of patients with LOH in the HLA region **(A)** Patient 10, **(B)** Patient 9 and **(C)** Patient 22. Lena: Lenalidomine; NHR: No Hematological Response; CHR: Complete Hematological Response; 5-Aza: 5-Azazitidine; CT: Chemotherapy; MRD: Minimal Residual Disease.

***Patient 10*** had isolated del(5q) MDS with Low-Risk IPSS-R (score of 2); no mutational studies were carried out at the diagnosis in April 2012. After 5 cycles of lenalidomide therapy with no hematologic response and high toxicity, this patient underwent RIC-Allo-HSCT from a related donor with compatibility at all HLA loci (10/10) in October 2012. The patient suffered engraftment failure in May 2014, and the mutational profile at that time predicted a good prognosis (*SF3B1*, VAF= 40%) (Supplementary Figure 1A). A second RIC-Allo-HSCT obtained a complete response with full chimerism [34]. In November 2016, the patient relapsed and progressed to a secondary AML (mixed chimera with 35% of recipient) and received 2 cycles of 5-azacitidine. In February 2017, the chronic hepatic, cutaneous and digestive-graft versus host disease (GVHD) of the patient worsened, followed by death due to multiple organ failure and chronic grade 4 GVHD one month later (Figure 1A).

**Figure 2:**
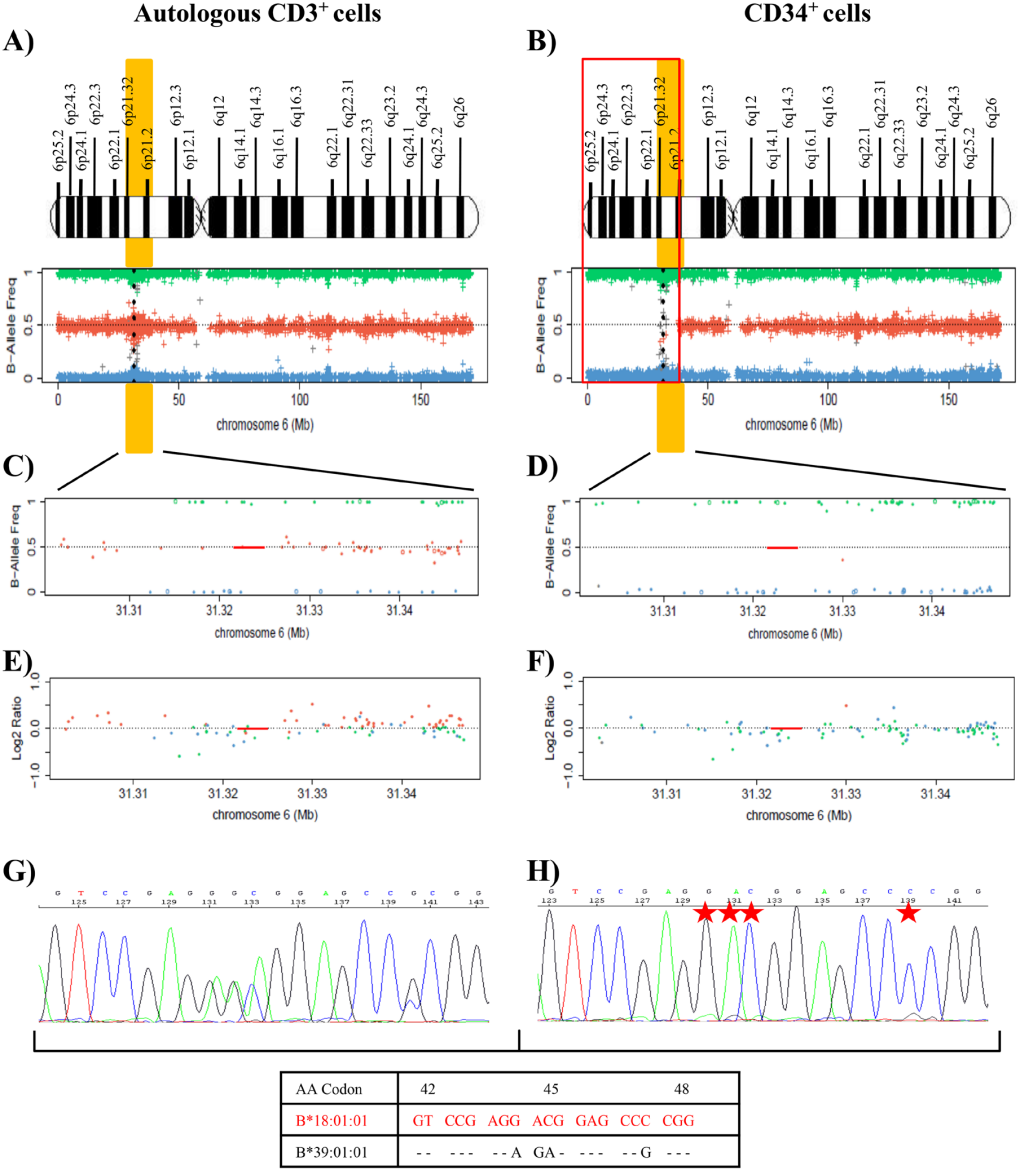
Results of SNP array on chromosome 6 and of the HLA Sanger sequencing of the patient 10 **(A)** and **(B)** These plots show the frequency of the B allele in CD3^+^ control cells **(A)** and CD34^+^ cells **(B)** in total chromosome 6, which indicates the allelic composition of each single-nucleotide polymorphism (SNP). BB genotypes have a B allele frequency of 1, AB genotypes a frequency of 0.5, and AA genotypes a frequency of 0. A schematic representation of chromosome 6 is included above the SNP array plots. HLA region is shown in chromosome 6p21.1-21.3 (yellow). Black points in SNP array plot indicate the position of the HLA-B locus in chromosome 6. A terminal deletion of 38 Mb (indicated in red) that involves HLA loci in CD34^+^ cells is detected in comparison to controls. **(C)** and **(D)** Plots for amplification of 6p21 region that involves HLA-B locus (red line), showing the frequency of the B allele in CD3^+^ control cells **(C)** and CD34^+^ cells **(D)**. SNP array results indicate a homozygosity pattern (genotypes AA=1 and BB=1) in CD34^+^ fraction. **(E)** and **(F)** Plots of logR ratio in CD3^+^ control cells **(E)** and CD34^+^ cells **(F)**, a measure of the copy number for each SNP. CD34^+^ cells conserved the copy number (logR ratio=0) along chromosome 6. The lack of heterozygous genotypes across the p arm of chromosome 6 in the absence of an alteration in copy number in the leukemic cells indicates CN-LOH. **(G)** and **(H)** Fragment of the sequencing electropherogram of exon 2 (codon 42 to codon 48) in the HLA-B locus of patient 10. In CD34^+^ cells **(H)** a loss of heterozygosity in polymorphic positions (red stars) in exon 2 in comparison to CD3^+^ cells **(G)**. The nucleotide sequence detected in CD34^+^ cells corresponds to HLA-B*18:01 allele (red), while allele HLA-B*39:01 is lost.

The cytogenetic features of leukemic cells (isolated del(5q)) and driver mutation (*SF3B1*, VAF=17%) were the same before the second RIC-Allo-HSCT as after the subsequent relapse (Figure 1A and Supplementary Figure 1B). No additional mutations were detected in sequenced genes. Chimera studies on CD34^+^ isolated cells obtained at the time of the second relapse showed that the leukemic cells all belonged to the patient (data not shown). SNP array analysis was carried out in DNA from CD34^+^ and CD3^+^ autologous cells, detecting LOH in the CD34^+^ fraction due to a deletion of 38 Mb (approximately from p25.2 to p21.2) involving a large part of the short arm of chromosome 6 that encompasses the HLA region (Figure 2). B-allele frequency (BAF) plots on CD34^+^ cells revealed a homozygosity pattern in the distribution of SNPs (BAF= 0 or 1) in the 6p region (Figure 2B and 2D) compared with the control sample, which showed a heterozygosity pattern (BAF= 0, 0.5 or 1) (Figure 2A and 2C). In addition, log2 ratio studies in CD34^+^ cells showed conserved CN (log2 ratio= 0) similar to that of control cells (Figure 2E and 2F). All these data suggested a CN-LOH in CD34^+^ leukemic cells due to acquired uniparental disomy (aUPD) mechanisms.

Genomic HLA typing was performed, based on Sanger Sequencing, in order to verify the finding of LOH in the HLA region of leukemic cells (Figure 2G and 2H). Comparison of the sequencing electropherogram of CD34^+^ cells with that of control cells revealed LOH at all polymorphic positions in exons 2, 3, and 4 of loci HLA-B, -C and -DRB1, retaining HLA-B*18:01, C*07:01 and DRB1*11:04 alleles. HLA-A and -DQB1 loci were homozygous in CD3^+^ control cells and were therefore not informative for the sequencing analysis. In conclusion, CD34^+^ cells of patient 10 lost HLA-B*39:01, C*12:03 and DRB1*11:01 alleles (Figure 2G and 2H). Subsequent HLA Luminex typing of leukemic blasts confirmed the HLA allele losses detected by sequencing analysis (Table 2).

**Figure 3:**
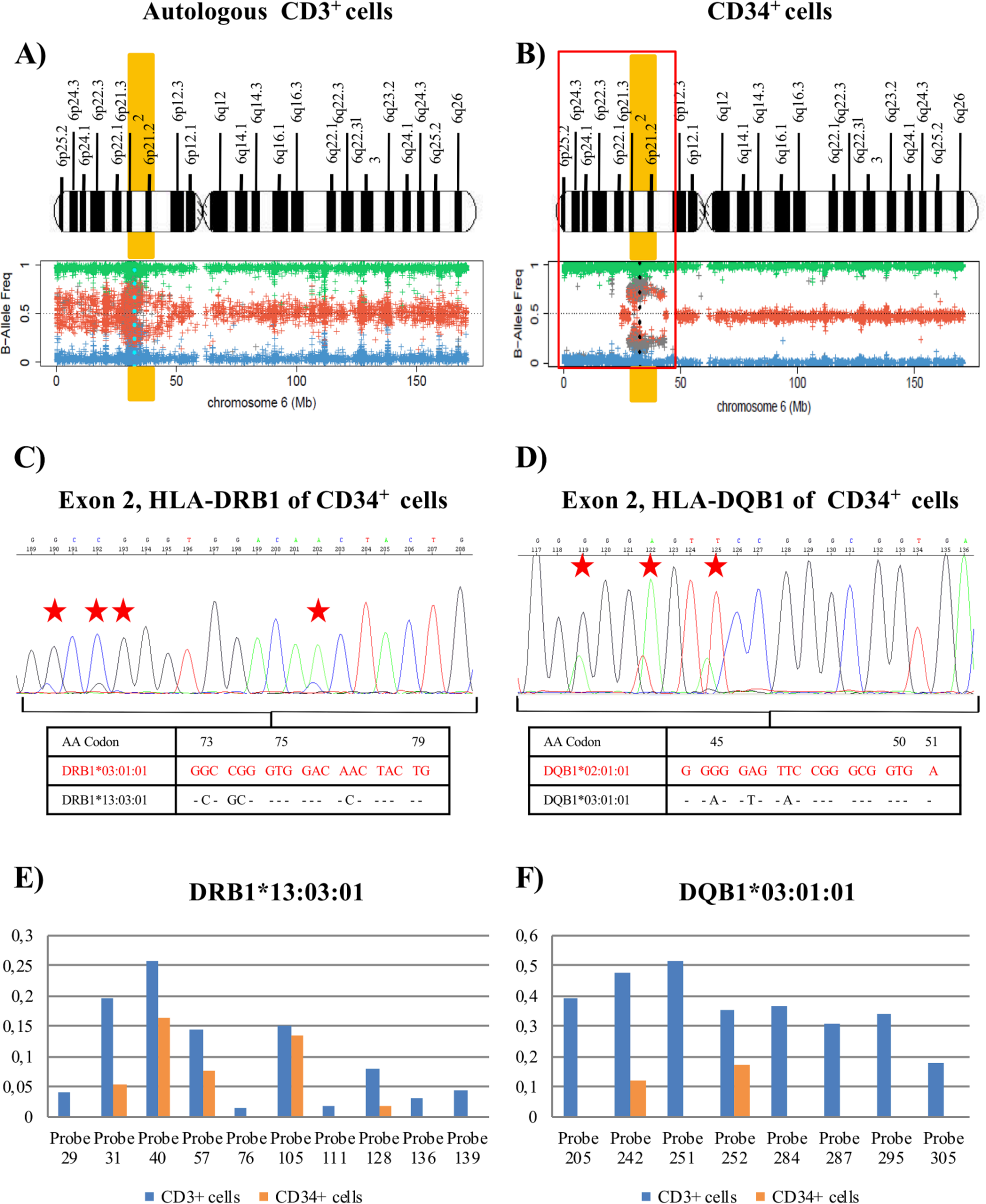
Results of SNP array on chromosome 6 and the HLA Sanger sequencing of the patient 9 **(A)** and **(B)** See Figure 2 footnotes for the interpretation of SNP array plots. Blue (CD3^+^ cells) and black (CD34^+^ cells) points in the SNP array plot of chromosome 6 show the position of the HLA-DRB1 locus in CD3^+^ control cells **(A)** and CD34^+^ cells **(B)**. We detected a terminal deletion of 40 Mb (indicated in red) that involves HLA loci in CD34^+^ cells. We detected a small region telomeric to the HLA loci that retained heterozygosity (Eliminate these words from the text). **(C)** and **(D)** Fragment of the sequencing electropherogram of exon 2 (codon 73 to codon 79) of HLA-DRB1 locus **(C)** and exon 2 (codon 44 to codon 51) of HLA-DQB1 locus **(D)** in CD34^+^ cells. The height of peaks at polymorphic sites (red stars) of the nucleotide sequence, corresponding to the DRB1*13:03 alleles and DQB1*03:01 alleles, was reduced in comparison to the signal intensity of the sequence for DRB1*03:01 and DQB1*02:01 alleles (red), suggesting that the LOH detected was not present in all leukemic blasts in the purified sample. **(E)** and **(F)** Representation of the difference between the adjusted median fluorescence intensity (MFI) value of the informative sequence-specific oligonucleotide (SSO) probes and the cut-off values for alleles **(E)** DRB1*13:03 and **(F)** DQB1*03:01 in CD34^+^ cells in comparison to CD3^+^cells, obtained for HLA typing using Luminex technology. The values represented were next or lower than the value of the cut-off in most SSO probes in CD34^+^ cells. The adjusted values for the different probes corresponded to: (MFI probe – MFI blank probe) / (MFI control probe – MFI blank control probe), according to manufacturer's instructions.

***Patient 9*** had isolated del(5q) MDS with Low-Risk IPSS-R (score of 2) (Table 1). Mutational studies were not carried out at the diagnosis in May 2013. After 36 cycles of lenalidomide therapy, a complete hematologic and cytogenetic response was observed [34]. In June 2016, the patient relapsed and progressed to sAML and finally died due to multiple organ failure. At the moment of the relapse/progression, no mutations were detected by NGS techniques. DNA from purified CD34^+^ and CD3^+^ autologous cells at the time of the relapse were used for SNP array analysis. Results revealed a complex pattern of LOH produced by a non-simultaneous double deletion affecting a region of 40Mb (approximately, from p25.2 to p12.3) that included the HLA loci in leukemic blasts compared with CD3^+^ cells (Figure 3A and 3B). This complex pattern is due to a small region telomeric to the HLA loci with heterozygosity retention, but this technique did not reveal its location at chromosomal level (Figure 3B). In addition, the Log2 Ratio plot showed a conserved copy number; therefore, this patient also had CN-LOH in CD34^+^ leukemic cells (data not shown).

SNP array results for HLA loci genotyping in CD34^+^ cells showed a lower amplification signal for SNPs associated with the alleles corresponding to the haplotype HLA-A*66:01; HLA-B*51:01; C*07:01; DRB1*13:03; DQB1*03:01, in comparison to the signal for haplotype HLA-A*30:02; HLA-B*18:01; C*05:01; DRB1*03:01; DQB1*02:01, a frequent haplotype in Spanish hematopoietic patients [35]. Next, analysis of Sanger sequencing electropherogram in the CD34^+^ cell fraction revealed in all polymorphic positions of the analyzed loci (HLA-DRB1 and -DQB1) a striking reduction in the height of the peaks that constituted the nucleotide sequence corresponding to the alleles HLA-DRB1*13:03 and DQB1*03:01 in comparison to the signal intensity of the sequence for alleles DRB1*03:01 and DQB1*02:01 (Figure 3C and 3D). These findings suggested that the haplotype loss might not be present in all purified cells of the CD34^+^ cell fraction, so that there would be a small proportion of pathological cells with heterozygosity retention, conserving the two HLA haplotypes. Likewise, the HLA typing analysis by Luminex technology did not assign DRB1*13:03 or DQB1*03:01 alleles in CD34^+^ samples, because most of the adjusted median fluorescence intensity (MFI) values were close to or lower than the cut-off value of the informative sequence-specific oligonucleotide (SSO) probes (Table 2 and Figure 3E and 3F).

***Patient 22*** had *de novo* AML (AML M1 FAB classification) with no cytogenetic anomaly; mutation studies were not carried out at the diagnosis in August 2014. After induction and two consolidation cycles of chemotherapy, a morphologic complete response (CR) and positive minimal residual disease (MRD) were recorded, and the patient underwent autologous transplantation in February 2015. The patient suffered a relapse in July 2017 and received second-line treatment with IdaFlag induction, obtaining CR with negative MRD. The patient then underwent RIC-Allo-HSCT from a related donor (10/10) in November 2017, obtaining a CR and full chimerism [34]. In January 2018 (52 days after RIC-Allo-HSCT), the acute hepatic, cutaneous and digestive-GVHD of the patient deteriorated, followed by death due to multiple organ failure and invasive pulmonary aspergillosis (Figure 1B).

**Figure 4:**
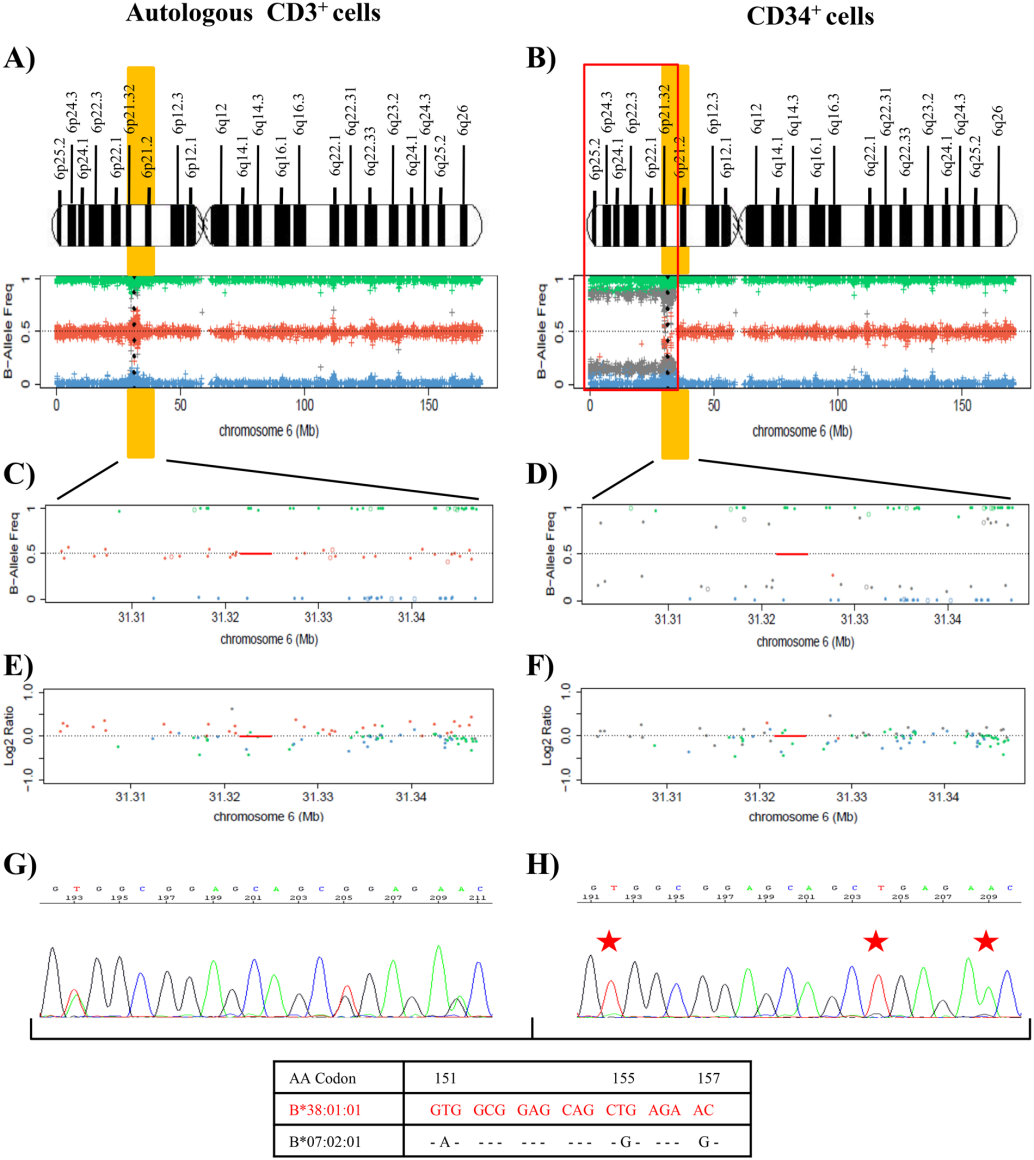
Results of the SNP array on chromosome 6 and the HLA Sanger sequencing of the patient 22 **(A)** and **(B)** See Figure 1 footnotes for the interpretation of SNP array plots. Black points in SNP array plots show the position of the HLA-B locus in chromosome 6. A terminal deletion of 36 Mb (outlined in red) that involve HLA loci in CD34^+^ cells is detected. **(C)** and **(D)** These plots are an amplification of 6p21 region that involves the HLA-B locus region (red line), showing the frequency of the B allele in CD3^+^ control cells **(C)** and CD34^+^ cells **(D)**. SNP array results indicate a homozygosity pattern (genotypes AA=1 and BB=1) in the CD34^+^ fraction. **(E)** and **(F)** Log2ratio at 0 indicates no copy number alteration and therefore a CN-LOH in CD34^+^ cells. **(G)** and **(H)** Fragment of the sequencing electropherogram of exon 3 (codon 151 to codon 157) of HLA-B locus. In CD34^+^ cells **(H)**, a loss of heterozygosity in polymorphic positions (red stars) in exon 3 is observed in comparison to CD3^+^ cells **(G)**. The nucleotide sequence lost in CD34^+^ cells corresponds to the HLA-B*07:02 allele, while the HLA-B*38:01 allele is retained (Red).

Leukemic cells at the relapse in July 2017 (post-autologous transplantation) were *FLT3-*TKD-positive (VAF=43%) with mutations in *NMP1* (VAF=34%) and *WT1* (VAF=47.2%) genes (Table 1, Supplementary Table 1 and Supplementary Figure 1C−1E). SNP array analysis of DNA from purified CD34^+^ and CD3^+^ autologous cells at this time showed LOH due to a deletion of 36 Mb (approximately from p25.2 to p21.2) that encompassed the HLA region in leukemic blasts in comparison to control cells. CD34^+^ cells showed a homozygotic pattern in the altered region with a conserved copy number, suggesting CN-LOH, which also affected the HLA region (Figure 4A−4F). Sanger sequencing studies of CD34^+^ cells demonstrated LOH in polymorphic positions in all exons at HLA class I and II loci (Figure 4G and 4H), retaining the HLA haplotype HLA-A*26:01; B*38:01; C*12:03; DRB1*01:03; DQB1*03:02 in comparison to the heterozygous CD3^+^ sample (A*02:01, A*26:01; B*07:02, B*38:01; C*07:02, C*12:03; DRB1*01:03, DRB1*04:02; DQB1*03:02, DQB1*05:01). HLA Luminex typing confirmed the loss of haplotype detected by sequencing analysis in blast cells *versus* control cells (Table 2).

## DISCUSSION

Alteration of HLA-I expression on the cell surface is frequently used by tumors to evade T-cell control [13, 36–38]. Various HLA-I phenotypes have been reported in tumors arising in different tissues, including total loss or downregulation of HLA-I antigens, HLA-haplotype, -locus or -allele loss, among others, and these changes have been attributed to multiple molecular mechanisms [13, 39, 40]. The most frequently cited mechanism involves LOH HLA, suggesting a common immune evasion strategy in cancers that may result from positive selection, according to recent studies [32, 41, 42]. This mechanism involves the presentation of a smaller repertoire of putative neoantigens to CD8^+^ T cells in comparison to heterozygous status, resulting in a less effective antitumor response by cytotoxic T (CD8) cells [32, 42–44].

Most investigations of HLA expression have been performed in solid tumors, detecting a higher frequency of HLA losses in comparison to hematological neoplasms, with reports of 90% in cervical cancer [45], 49% in lung carcinoma [30, 32], and 20-70% in both melanoma [39, 46, 47] and laryngeal carcinoma [48]. It has been reported that partial loss of HLA alleles is a more frequent mechanism than total haplotype loss in some hematological neoplasms [23, 49]. In the present study of 27 patients, loss of a full HLA haplotype was detected in three (11%), and we provide the first report of LOH HLA in two patients (patient 10 and patient 22) who relapsed after identical-HSCT. Intriguingly, both relapsing patients had a favorable clinical-biological profile, with no risk factors such as HMR mutations or complex karyotypes. One of them (patient 10) had isolated del(5q) with a low risk according to the IPSS/IPSS-R system and good karyotype (low cytogenetic risk); the same *SF3B1* mutation was detected before the second HSCT as at the subsequent relapse/AML transformation, with no additional mutations. *SF3B1* is a molecular marker of a good prognosis and has been associated with positive post-HSCT outcomes [9]. The other patient (Patient 22) had a *de novo* AML without cytogenetic anomaly, and the mutations detected at the time of relapse (*FLT3-TKD, NPM1, WT1*) have not been considered molecular markers of an unfavorable prognosis [50].

The remaining patient with LOH HLA (patient 9) was diagnosed with sAML and, in common with patient 10, had a favorable clinical profile. It is likely that the selective HLA loss in these patients favors immune evasion and may explain the expansion and proliferation of the malignant clone. In this line, a recent study observed an increased homozygosity rate at HLA-A, B, C and DRB1 loci in chronic lymphocytic leukemia patients in comparison to the general population, suggesting LOH HLA as a possible mechanism that evolves through positive selection [51]. Furthermore, in a study of patients with aplastic anemia, the finding of CN-LOH at 6p arms involving the HLA region was described as an escape mechanism from CTL autoimmunity [25].

In contrast, we found no case of LOH HLA in patients with a high risk of progression to leukemia according to their IPSS and IPSS-R scores, the presence of HMR mutations, and their complex karyotypes. It is likely that a proliferative advantage from the accumulation of these risk factors accounts for the disease progression, although other immune evasion mechanisms might be involved. Alternatively, it has been found that the role of cellular immune responses significantly differs between low- and high-risk (IPSS-classified) MDS. Low-risk MDS is associated with autoimmune disease-like characteristics, increased NK cell levels, activation of CTLs, reduced regulatory T-cell (Treg) count, and increased type 17 T-helper (Th17) cell count. In contrast, high-risk MDS bone marrow is characterized by a microenvironment that suppresses immune responses through the presence of dysfunctional NK cells, reduced and exhausted CTLs, and higher levels of Tregs and immunosuppressive cytokines. We propose that certain features of the tumor microenvironment in low-risk MDS patients permit immunoediting of the cancer cells, which thereby acquire a weakly immunogenic phenotype (HLA loss) that facilitates immune escape [52].

Haplotype loss in chromosome 6 has been reported in patients after haploidentical-HSCT, with findings of the loss of mismatched HLA by the aUPD mechanism in around 20% of relapsing patients [27, 28, 53]. The fact that leukemic cells are in direct contact with NK cells in myelodysplastic syndromes and other hematological disorders may explain why complete loss of HLA alleles has rarely or never been observed in these diseases. No case of HLA-I cell surface total loss was detected in the present study (data not shown). Neoplastic cells are less exposed to the action of NK cells in solid tumors, for which various immune escape mechanism have been reported, including homing defects/difficulties that exclude NK cells from direct contact with cancer cells [54–56]. Our group found that tumor nests in HLA-I negative NSCLC cases are poorly infiltrated by CTLs and NK cells [41]. Progressive alteration in the phenotype of NK cells from healthy tissue to tumor tissue has also been described, with the emergence of a non-cytotoxic phenotype in tumor tissue [53–57]. Interestingly, defects in NK cell function have also been described in myelodysplastic syndromes, mostly attributed to the unsuccessful or inadequate generation of mature/functionally competent NK cells, which might contribute to disease progression through impaired immune surveillance [58, 59]. Our tumor microenvironment study in bone marrow samples from MDS patients detected striking alterations in the functional phenotype of NK cell populations (data not shown). Notably, the patient with sAML and partial LOH HLA (not present in the total purified CD34^+^ cell fraction) had HLA-C alleles belonging to different C-groups (HLA-C*07:01 (C1 group) and HLA-C*05:01 (C2 group)) that are suppressor ligands of killer immunoglobulin like-receptor (KIR) in NK cells. In this context, a single haplotype loss in neoplastic cells would involve attack by NK cells, given that the KIRs would not interact with the HLA-C antigens lost. In addition, LOH HLA was restricted to HLA-B alleles that belonged to the HLA-Bw6 group, whereas the HLA-Bw4 group was conserved, and HLA-Bw6 loss has been described as an escape mechanism not only from CTLs but also from NK cells [60].

In contrast, the two patients with LOH HLA after identical-HSCT had the same two HLA-C antigens (HLA-Cw7, Cw12) belonging to the C1 group. LOH HLA may take place, at least in patients with permissive phenotype (inhibitory ligands of the same C-group), with tumor cells becoming invisible to T and NK cell attack. These results also explain why downregulation of some alleles, but not complete loss of HLA class I antigen expression, is observed in leukemias prior to transplant and may lead to the escape from immune surveillance and adversely impact clinical outcome [61]. In fact, mutations in the gene that encodes the ß2-microglobuline chain have not been described in hematological neoplasms with peripheral expression, but have been reported in patients with lymphomas [62]. A likely explanation is that complete loss of HLA class I antigen expression renders cells susceptible to NK cell-mediated killing, whereas partial loss of HLA class I alleles might protect the tumor cell from both a T cell- and NK cell-mediated immune response [13, 29, 63, 64].

In conclusion, in the absence of mechanisms that satisfactorily explain the aggressive behavior of the disease in the three patients described in this paper, who all had favorable clinical and mutational profiles, these data suggest that LOH HLA may be an important immune evasion mechanism that allows clonal evolution of the disease. Furthermore, the study of LOH HLA may help to explain poor clinical outcomes in apparently low-risk patients.

## MATERIALS AND METHODS

### Patients

The study included 27 patients from Granada region in Spain diagnosed between December 2016 and February 2018. Patients were classified according to the WHO-2016 classification [33]. Eight patients (6 males and 2 females, mean age 73 years) were diagnosed with MDS, including one with excess blasts-1 (MDS EB-1) and seven with excess blasts-2 (MDS EB-2); eleven (8 males, 3 females; mean age 73 years) were diagnosed with AML secondary to MDS (sAML), two (78-yr-old male and 66-yr-old female) with chronic myelomonocytic leukemia (CMML), and six (3 males, 3 females; mean age 59 years) with *de novo* AML. Patient characteristics are exhibited in Table 1. All patients signed informed consent to participate in the study, which followed the principles of the Helsinki Declaration and was approved by the ethical committee of our hospital.

### Automated CD34^+^ and CD3^+^ cells isolation

An automatic immunomagnetic cell processing system (autoMACS Pro, Miltenyi Biotec) was used to isolate CD34^+^ and CD3^+^ cells from the bone marrow and peripheral blood of patients. Peripheral blood mononuclear cells (PBMCs) were isolated by Ficoll-Hypaque centrifugation (GE Healthcare Bio-Sciences). CD34^+^ cells were isolated with a CD34^+^ cell isolation kit (MicroBead Kit, human, Miltenyi Biotec), according to the manufacturer's instructions. MACS buffers (Miltenyi Biotec) were used for incubation with beads and for cell separations on the AutoMACS Cell Separator. CD3^+^ cells isolation was performed using CD3^+^ cell isolation kit (MicroBead Kit, human, Miltenyi Biotec) following the same methodology as the isolation of CD34^+^ cells. Flow cytometry was used to evaluate CD34^+^ and CD3^+^ purity. All samples showed purity ≥ 96%.

### Cytogenetics

Fluorescence *in situ* hybridization (FISH) techniques were used to evaluate the principal chromosomical alterations in MDS (del(5q), del(7q), del(20q) and 8 trisomy. Analyses were performed using commercially available probes (LSI 5q31 (EGR1); LSI 7q31 (D7S522); LSI 20q12 (D20S108); CEP 8) (Abbott) using conventional metaphase techniques. Karyotyping was performed in an external laboratory.

### Flow cytometry analysis

The effectiveness of CD34^+^ cell separation was determined by 8-color flow cytometry analysis on a FACSCantoII (BD Biosciences, New Jersey). CD34^+^ cells (20 μl) were incubated with anti-CD45 (CD45V_500_), anti-CD117 (CD117PE-CY^™^ 7), anti-HLA-DR (HLA-DRV_450_), and anti-CD3 (CD3APC-H7) monoclonal antibodies (mAbs) (BD Biosciences, San Diego, CA) for 20 min at room temperature. After centrifugation and washing with phosphate saline (PBS), 200 μl of FACS Lysing Solution (BD Biosciences) were added. Data were analyzed using Infinicyt 10.0 software (Cytognos).

### DNA isolation

Genomic DNA was obtained from peripheral blood and bone marrow samples or from CD34^+^- and CD3^+^-purified cells using a QIAamp DNA Blood Mini Kit (QIAGEN). Extracted DNA was quantified using a Qubit dsDNA BR Assay Kit (ThermoFisher Scientific, Walltham, MA) and Qubit 2.0 Fluorometer.

### Next-generation sequencing (NGS) of myeloid gene panel

The mutational profile of the patients was studied by NGS using a commercial gene panel (TruSight Myeloid Sequencing Panel, Illumina, San Diego, CA) that includes 54 myeloid target genes. Amplicon sequencing libraries were prepared from 50 ng of DNA per sample using TruSeq Custom Amplicon Assay and TruSight Myeloid Sequencing Panel Oligos. Libraries were normalized to 4nM and pooled in groups of 8 patient libraries. Paired-end sequencing (2x150 cycles) of each library pool was performed on a MiSeq platform with a reagent kit V3 (Illumina, San Diego, CA) following the manufacturer's instructions. The fastq files obtained after sequencing were loaded in the Sophia Genetics application (version 4.6.2) for sequence alignment, variant annotation and subsequent analysis. Integrative Genomics Viewer version 2.3.68 (Broad Institute, Cambridge, MA) was used to visualize read alignment data.

### Single nucleotide polymorphism (SNP) array analysis

DNA samples from leukemic cells and controls (autologous CD3^+^ cells) were genotyped using the Illumina Infinium assay on the Immunochip (v2), following the manufacturer’s instructions (Illumina, San Diego, CA), which detects 253,703 SNPs selected according to the GWAS of immune system diseases. Illumina Genome studio software was used to obtain data on the loss of heterozygosity (LOH) and copy number (CN), expressed as "theta" and "R" values, respectively, with "theta" representing the B-allele frequency and "R" the combined fluorescence intensity of both channels. "Theta" can be interpreted directly to detect LOH using BCF tools [PMID:26826718], while "R" must be compared with a reference standard to detect regions of CN loss or gain. In the present study, this standard was based on the median fluorescence value per probe in immunochip data from 1632 non-cancer samples of European ancestry, subsequently obtaining log-ratios. A log-ratio distribution around zero can be regarded as neutral CN, while chromosomal intervals of mainly positive (or negative) log-ratios can be interpreted as CN gain (or loss). Chromosomal stretches of B-allele frequencies with values of mainly zero or one can be interpreted as LOH.

### HLA genomic typing by luminex technology

DNA from CD34^+^ cells and autologous CD3^+^ lymphocytes were used to perform HLA genomic typing with the LIFECODES HLA-A, -B, -C, -DRB1 and -DQB1 Typing Kits–Rapid (IMMUCOR, Georgia) following the manufacturer’s instructions. The Luminex 100/200^™^ System, based on xMAP Technology (Luminex®, Austin, Texas) and the Match-It DNA v1.2 software (IMMUCOR) were used to analyze HLA typing, enabling detection of haplotype, locus or allele losses in HLA genes in leukemic cell samples.

### HLA genomic typing by sanger sequencing

Sanger sequencing analysis was performed with the GenDxAlleleSEQR kits (GENDX, Utrecht) to confirm the results obtained by SNP-array, using DNA from CD34^+^ cells and autologous CD3^+^ T cells. CE-marked SBTengine® software was used for high resolution analysis of HLA sequencing data.

## SUPPLEMENTARY MATERIALS FIGURE AND TABLE


