# Supplementary material for: Genomic loss of HLA alleles may affect the clinical outcome in low-risk myelodysplastic syndrome patients
Source: Oncotarget. 2018 Dec 11;9(97):36929–44. doi: 10.18632/oncotarget.26405 (PMC6319343; doi:10.18632/oncotarget.26405)
Supplement: Supplementary file 1 [file oncotarget-09-36929-s001.pdf]

Genomic loss of HLA alleles may affect the clinical outcome in low-risk myelodysplastic syndrome patients

SUPPLEMENTARY MATERIALS

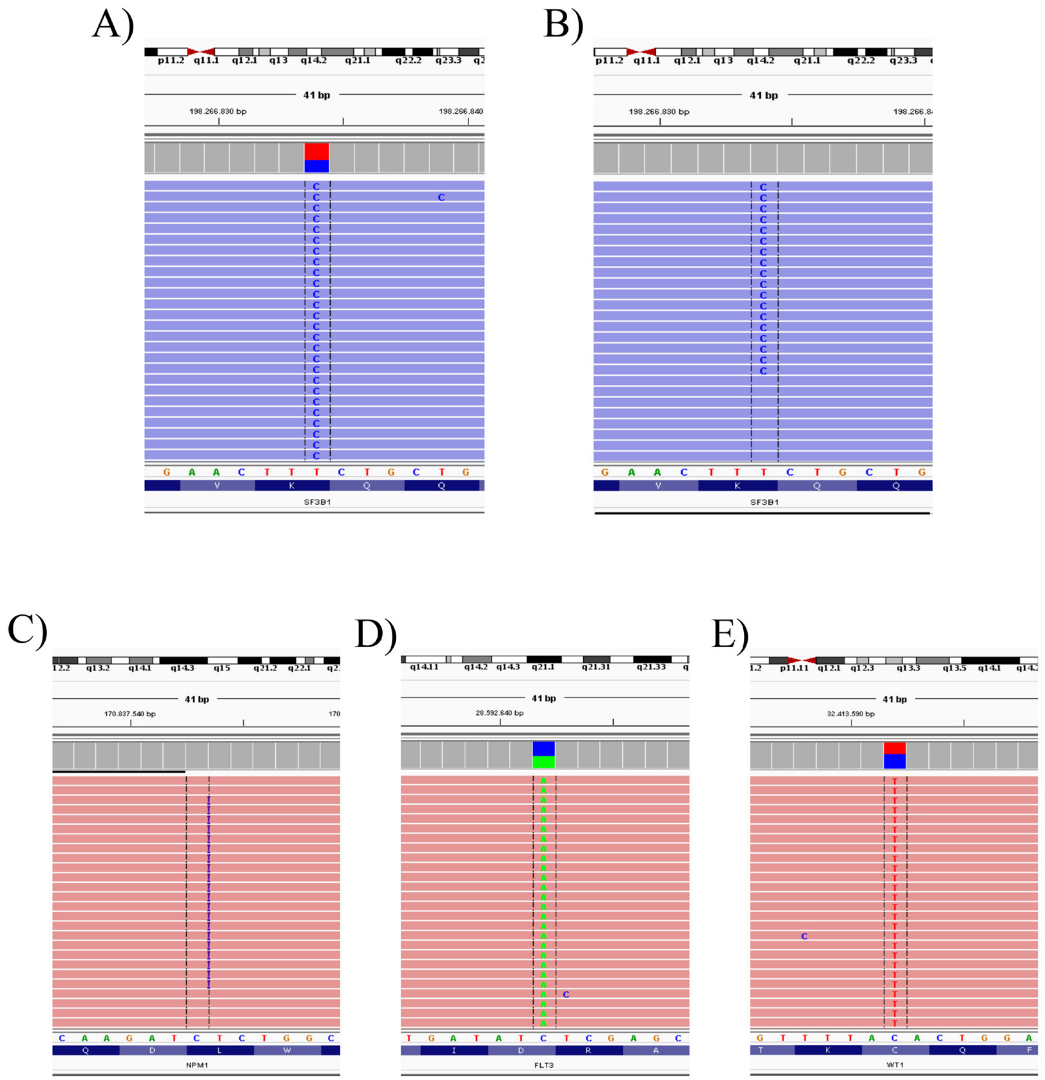

Supplementary Figure 1: Integrated Genomic Viewer (IGV) screenshot of the gene variants detected in patients 10 **(A** and **B)** and 22 **(C, D** and **E)** using NGS techniques. **(A)** SF3B1 variant found in patient 10 at the time of the second HSCT consisted in a recurrent missense mutation (c.2098A>G) in exon 15 which resulted in an amino acid change at position 700 (p.Lys700Glu). The total coverage at the position was 7221 reads, with 40% variant allele frequency (VAF) (2888 reads with SF3B1 mutation variant). **(B)** At the time of relapse and progression to sAML in patient 10, the same SF3B1 mutation variant (c.2098A>G) was detected. The VAF was 17% with total coverage at the position of 2870 reads (488 reads with SF3B1 mutation variant). No additional mutations were detected at this stage of the patient's disease. In patient 22, NPM1 **(C),** FLT3 **(D)** and WT1 **(E)** mutations were detected at the time of the relapse. **(C)** NPM1 variant was a recurrent mutation type A that consisted in a 4 bp insertion (c.860_863dupTCTG) resulting in a frameshift alteration (p.Trp288Cysfs*). The mean coverage of sequence was 5400 reads, with 34% VAF (1836 altered reads). **(D)** FLT3 variant was the missense mutation in exon 20, which resulted in an amino acid change at position 835 (p.Asp835Tyr) (FLT3-TKD mutation). The total coverage at the position was 15300 reads, with 43% VAF (6579 reads with FLT3-TKD mutation). **(E)** WT1 variant consisted in a missense mutation (c.1307G>A) resulting in an amino acid change at position 436 (p.Cys436Tyr). The VAF was 47% with total coverage at the position of 8960 reads (4211 reads with WT1 mutation variant)**.**

Supplementary Table 1: Description of karyotypes and mutational profiles for 27 patients in the study.

PATIENTS	WHO-2016	KARYOTYPE DESCRIPTION	MUTATIONAL PROFILE			
			Gen	Nucleotide	VAF (%)	Protein
1	*MDS EB-1*		***TP53***	*c. 659A>G*	*84*	*p.Tyr220Cys*
			TET2	*c.3635T>A*	*84*	*p.Leu1212**
			U2AF1	*c.470A>G*	*3*	*p.Gln157Arg*
2	MDS EB-1	44,XY, -5, -7	***TP53***	c. 455C>T	47	p.Pro152Leu
3	MDS EB-2	45,XY, -7(4)/46,XY(16)	ND	ND	ND	ND
4	MSD EB-2	45,X, del(11)(q21;q23)	***RUNX1***	c.86T>C	50	p.Leu29Set
5	MSD EB-2	46,XX, add(6), add(10), del(11), add(14), -15, -19, +1mar, +2mar	Not detected			
6	MDS EB-2	ND	***ASXL1***	c.1934dupG	42	p.Gly646Trpfs*12
			RUNX1	*c.519C>T*	*41*	*p.Arg177**
			SRSF2	*c.284C>T*	*49*	*p.Pro95Leu*
			IDH1	*c.395G>A*	*36*	*p.Arg132His*
			TET2	*c.1648C>T*	*2*	*p.Arg550**
7	MSD EB-2	46,XY, del(5)(q13;q33) (8)/45,XY, -1, del(5), add(10)(q26) (23)/49,XY, +1, del(5), +11, +14 (8)	***TP53***	c.742C>T	89	p.Arg248Trp
8	MSD-EB-2	46,XX, del(5q)(q15;q33)	SF3B1	c.2098A>G	33	p.Lys700Glu
			NPM1	*c.860_863dupTCTG*	*26*	*p.Trp288Cysfs**
9	sAML	46,XX, del(5q)	Not detected			
10	sAML	46,XX, del(5q)	SF3B1	c.2098A>G	17	p.Lys700Glu
11	sAML	45,XY, -3, del(5) (q13;q33), add(6)(p25), +8, add(17)(p13), del(20) (q11.2), -22	***TP53***	c.844C>T	59	p.Arg282Trp
12	sAML	46,XX	SF3B1	c.2098 A>G	49	p.Lys700Glu
			DNMT3A	*c.2612C>T*	*45*	*p.Pro871Leu*
			TET2	*c.4570C>T*	*51*	*p.Gln1524**
13	sAML	47,XY, +8	***ASXL1***	c.1934dupG	42	p.Gly646Trpfs*12
			EZH2	*2003A>G*	*50*	*p.Asn668Ser*
			ETV6	*c.1079G>C*	*13*	*p.Trp360Ser*
			KRAS	*c.35G>T*	*29*	*p.Gly12Val*
			PTPN11	*c.1508G>A*	*7*	*p.Gly503Glu*
			ABL1	*c.820G>A*	*10*	*p.Glu274Lys*
14	sAML	ND	***TP53***	c.638G>T	67	p.Arg213Leu
			U2AF1	*c.467G>A*	*40*	*p.Arg156His*
			NRAS	*c.35G>C*	*11*	*p.Gly12Ala*
15	sAML	ND	***RUNX1***	c.1016_1017insGT	60	p.Ile339Metfs*
			U2AF1	*c.101C>T*	*42*	*p.Ser34Phe*
			BCOR	*c.4871_4872delAG*	*43*	*p.Gln162Argfs*13*
			NRAS	*c.356G>A*	*22*	*p.Gly12Asp*
			FLT3-TKD	*c.2503G>A*	*13*	*p.Asp835Asn*

PATIENTS	WHO-2016	KARYOTYPE DESCRIPTION	MUTATIONAL PROFILE			
			Gen	Nucleotide	VAF (%)	Protein
16	sAML	ND	***RUNX1***	c.404G>A	9	p. Lys580Glu
			EZH2	*c.1975G>A*	*41*	*p.Asp659Asn*
			ASXL1	*c.2122C>T*	*44*	*p.Gln708**
			SF3B1	*c.2342A>G*	*44*	*p.Asp781Gly*
			SETBP1	*c.2608G>A*	9	*p.Gly870Ser*
17	sAML	ND	RUNX1	c.416G>A	44	p.Arg139Gln
			SF3B1	*c.2098A>G*	*46*	*p.Lys700Glu*
			IDH2	*c.419G>A*	*46*	*p.Arg140Gln*
			DNMT3A	*c.2645G>A*	*46*	*p.Arg882His*
18	sAML	ND	***TP53***	c.1043delT	57	p.Leu348Trpfs*22
19	sAML	ND	***RUNX1***	c.792_803del10ins2	48	p.Ile264Metfs*17
			EZH2	*c.857G>A*	*47*	*p.Cys286Tyr*
			EZH2	*c.619C>T*	*48*	*p.Arg207**
			IDH2	*c419G.>A*	*48*	*p.Arg140Gln*
			CUX1	*c.764A>T*	*47*	*p.Glu255Val*
20	CMML-0	46, XY	STAG2	c.226C>T	90	p.Gln76*
			ASXL1	*c.1773C>A*	*51*	*p.Tyr591**
			SRSF2	*c.284C>A*	*51*	*p.Pro95His*
			TET2	*c.3732_3733del CT*	*50*	*p.Tyr1245Glyfs*22*
			TET2	*c.3733_3737delTACTC*	*43*	*p.Tyr1245Glyfs*21*
21	CMML-1	46,XX, del(5q)	***TP53***	*c.393_395delCAA*	*97*	*p.Asn131del*
			KRAS	c.35G>T	16	p.Gly12Val
22	De novo AML	45, X (5) / 46, XY (15)	NPM1	c.860_863dupTCTG	34	p.Trp288Cysfs*
			FLT3	*c.2503G>T*	*43*	*p.Asp835Tyr*
			WT1	*c.1307G>A*	*47*	*p.Cys436Tyr*
23	De novo AML	46, XY	Not detected			
24	De novo AML	47, XY, +8	NPM1	c.860_863dupTCTG	29	p.Trp288Cysfs*
			PTPN11	*c.205G>A*	*10.4*	*p.Glu69Lys*
25	De novo AML	ND	ZRSR2	c.1354C>T	51	p.Arg452Cys
26	De novo AML	46, XX, del(5)(q13;q33)	DNMT3A	c.2711C>T	50	p.Pro904Leu
27	De novo AML	ND	**TP53**	c.488A>G	46	p.Tyr163Cys
			TP53	*c.919+1G>A*	*42*	

VAF: Variant Allele Frequency; ND: No Data.

Genes in bold are High Molecular Risk (HMR) genes [1].

REFERENCES

1. Bejar R, Steensma DP. Recent developments in myelodysplastic syndromes. Blood. 2014; 124:2793-803. https://doi.org/10.1182/blood-2014-04-522136.
